# Variola Ten Days after Successful Vaccination

**Published:** 1871-02

**Authors:** 


CASE OF VARIOLA TEN DAYS AFTER SUCCESSFUL VACCINATION. —An infant, 27 days old, having every appearance of health, was brought to the Hospice des Enfants-Assistés on February 28, and vaccinated next day. On March 8, on account of the perfection of the pustules and the vigor of the child, twenty nuns, fifteen nurses, and a ladies' boarding school were all revaccinated from it. The next day an eruption appeared, which proved to be variola, of which the child died on March 13. None of those vaccinated from it took smallpox. In several the revaccination succeeded.—*Revue Med.*, Sept. 3.

